# Low-intensity pulsed ultrasound decreases major amputation in patients with critical limb ischemia: 5-year follow-up study

**DOI:** 10.1371/journal.pone.0256504

**Published:** 2021-08-19

**Authors:** Farina Mohamad Yusoff, Masato Kajikawa, Takayuki Yamaji, Yuji Takaeko, Yu Hashimoto, Aya Mizobuchi, Yiming Han, Shinji Kishimoto, Tatsuya Maruhashi, Ayumu Nakashima, Yukihito Higashi

**Affiliations:** 1 Department of Cardiovascular Regeneration and Medicine, Research Institute for Radiation Biology and Medicine, Hiroshima University, Hiroshima, Japan; 2 Division of Regeneration and Medicine, Medical Center for Translational and Clinical Research, Hiroshima University Hospital, Hiroshima, Japan; 3 Department of Cardiovascular Medicine, Hiroshima University Graduate School of Biomedical Sciences, Hiroshima, Japan; 4 Department of Stem Cell Biology and Medicine, Hiroshima University Graduate School of Biomedical Sciences, Hiroshima, Japan; Osaka University Graduate School of Medicine, JAPAN

## Abstract

Various therapeutic strategies for angiogenesis are performed to improve symptoms in patients with critical limb ischemia (CLI). Pre-clinical studies have shown that low-intensity pulsed ultrasound (LIPUS) exposure induces angiogenesis. LIPUS may be a new stratergy for treatment of CLI. The purpose of this pilot trial was to evaluate outcomes in patients with CLI who were treated with LIPUS. Fourteen patients with CLI, who were not candidates for angioplasty or surgical revascularization, were enrolled in this study. Historical control data were obtained from the Hiroshima University PAD database. The primary endpoints were major amputation and death. The outcomes were compared in 16 lower limbs of the 14 patients with CLI who were treated with LIPUS and in 14 lower limbs of 14 patients with CLI as historical controls. All patients were followed for after 5 years after treatment with LIPUS. The mean duration of LIPUS exposure in the LIPUS group was 381± 283 days. During the 5-year follow-up periods, there were 3 major amputations and 7 deaths in the LIPUS group and there were 14 major amputations and 7 deaths in the historical control group. The overall amputation-free survival rate was significantly higher in patients who were treated with LIPUS than in historical controls. There was no significant difference between overall mortality-free survival rates in the LIPUS group and historical control group. LIPUS is a noninvasive option for therapeutic angiogenesis with the potential to reduce the incidence of major amputations in patients with CLI.

## Introduction

Critical limb ischemia (CLI) has been considered as the end-spectrum of peripheral artery disease (PAD) and manifests as chronic (>14 days) inadequate tissue perfusion with or without tissue loss. Patients with CLI are at a high risk for limb amputation, cardiovascular complications, and death [1,2]. A large number of patients with CLI are at risk for major amputations due to the increasing number of PAD patients worldwide. Management of PAD and specific interventions for macrovascular lesions with limb preservation revascularization options are addressed in guidelines [3,4].

It was reported that one third or more of patients with CLI were deemed “no-option” at the time of diagnosis and opted for limb amputation [5]. There have been diverse degrees of consideration in giving the option of amputation to patients with CLI. The cost of care, re-hospitalization, re-interventions and rehabilitation after limb amputation has been a global economic burden. All of these factors significantly impair the quality of life for patients with CLI by imposing a substantial burden on a patient’s emotional, social, and physical well-being [6]. With a better understanding of the pathogenesis of microvasculature in PAD [7–10], a disease with not only macrovascular involvement, but also widespread microvascular involvement, improvement in precision medicine will enable limb amputation to be avoided in more patients and will improve the quality of life.

Pre-clinical studies have shown that LIPUS exposure induces angiogenesis [11–14]. Through stimulation of angiogenic factors such as interleukin-8, basic fibroblast growth factor (bFGF) and vascular endothelial growth factor (VEGF), via the extracellular signal-regulated kinase (ERK)/Akt/endothelial nitric oxide synthase/VEGF pathway, LIPUS exposure has the potential to provide beneficial cellular therapeutic effects on limb ischemia by inducing microvascular regeneration and to improve clinical symptoms in patients with CLI and reduce the risk of amputation [14]. In addition, LIPUS has also been investigated and used to facilitate bone fracture healing by osteogenesis in humans [15–17].

The present study was performed to evaluate 5-year outcomes in atherosclerotic PAD patients with CLI who were treated with LIPUS.

## Materials and methods

### Study patients

Fourteen atherosclerotic PAD patients with CLI were diagnosed after they presented with severe rest pain with or without non-healing ulcers and were not candidates for angioplasty or surgical revascularization. These patients were enrolled for a LIPUS clinical trial from 2011 to 2015. For historical control analyses, data were obtained from the Hiroshima University PAD database. Rheumatoid factor, lupus anticoagulants, and serological investigations were assessed to rule out other vasculitis and hypercoagulable states. The diagnosis of arterial occlusion leading to ischemia was confirmed by angiography. CLI was classified according to the guidelines of the TransAtlantic Inter-Society Consensus II (TASC II) [2]. Major amputation is defined as above-the-ankle amputation. The outcomes were compared in the 16 treated limbs of 14 patients in the treated group and in 14 patients with 14 affected limbs in the historical control group up to December 2020. The study protocol was approved by the Ethics Committee of Hiroshima University Graduate School of Medicine. Written informed consent for participation in the study was obtained from all patients during the progress of the clinical trial and for subsequent data acquisitions.

### Study design

This pilot trial was a retrospective, observational study to determine the 5-year outcomes of LIPUS in CLI patients, and data were obtained for determining the overall survival and major amputation-free survival rates of the patients was obtained. Reports of 3-point major adverse cardiovascular events (MACE) (myocardial infarction, stroke, and death from cardiovascular causes) and any causes of deaths in patients after 5 years of follow-up were also acquired. Amputation-free survival rates and overall survival rates for patients with CLI who were treated with LIPUS were compared to those of historical controls. The UMIN-CTR Clinical Trial ID is UMNI000004901.

### Historical controls

Historical control data were obtained from the Hiroshima University Hospital PAD database. These atherosclerotic PAD patients with CLI were previously treated according to the standard practice in Hiroshima University Hospital between 2011 to 2015 and had limbs (“no-option”) that were deemed unsalvageable and/or deemed for amputation during a 5-year follow-up period.

### LIPUS

**Fig 1:**
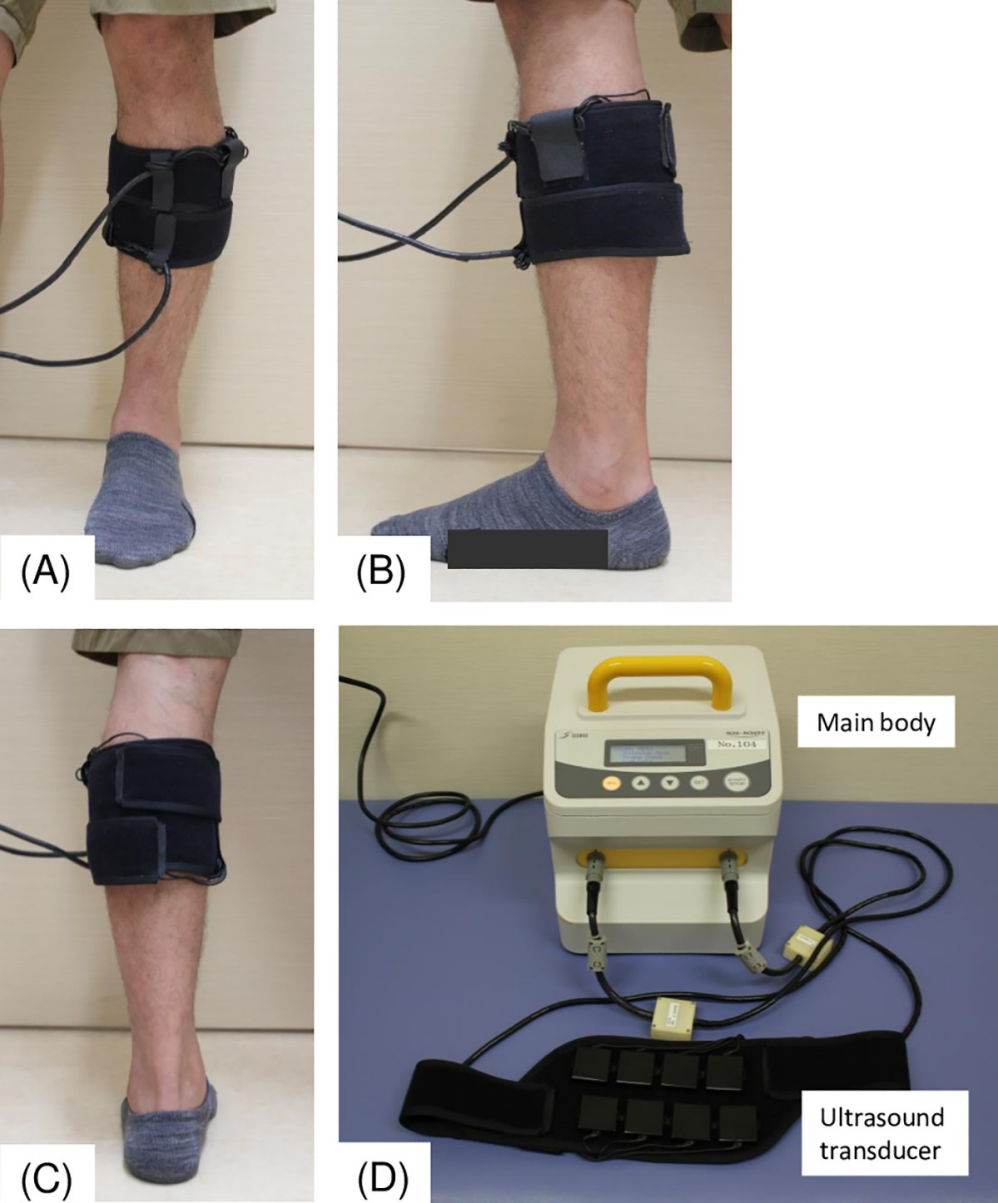
The LIPUS system set-up. (A) Application of transducers for LIPUS exposure in the gastrocnemius of the ischemic leg (anterior view). (B) Application of transducers for LIPUS exposure in the gastrocnemius of the ischemic leg (lateral view). (C) Application of transducers for LIPUS exposure in the gastrocnemius of the ischemic leg (posterior view). (D) Appearance of the low-intensity pulsed ultrasound (LIPUS) device.

The LIPUS device (SX-1001, Nippon Sigmax Co. Ltd., Tokyo, Japan) with a transducer element (Nippon Sigmax Co. Ltd.) emits ultrasound frequency of 2 MHz ± 10%, ultrasound output power of 30 mW/cm^2^, beam nonuniformity ratio of 5 ± 2, pulse duration of 200 μ ± 5%, pulse repetition frequency of 1 kHz ± 5%, pulse duty of 20% ± 5% and output duration of 20 minutes per probe. Ultrasound exposure was applied over the skin of the calf region of the symptomatic limb for 20 min per day and the subject could be either in a supine or seated position. The gap between the transducer and the skin was filled with ultrasonic gel. Eight transducer probes were attached to the symptomatic lower limb. The LIPUS device is portable and was used by patients at home with recorded log data [14] (Fig 1).

### Statistical analysis

Results are presented as frequencies for categorical variables and means±SD. All reported probability values were 2-tailed. Values of *P*<0.05 were considered significant. Continuous variables were compared by using ANOVA for multiple groups and the t-test between two groups. Categorical variables were compared by means of the chi-squared test or Fisher’s exact test. Time-to-event end-point analyses were performed by using the Kaplan-Meier method. The log-rank test was used to compare amputation-free survival and overall survival rates in the groups. Data were processed using JMP version 13.0 software (SAS Institute Cary, NC, USA).

## Results

### Clinical characteristics

Baseline clinical characteristics in the LIPUS group and the historical control group are summarized in Table 1. The prevalence of hypertension was significantly higher in the historical control group than in the LIPUS group. There were no significant differences in other parameters between the two groups.

**Table 1:** Clinical characteristics of patients with CLI who were treated with LIPUS and historical controls.

Variable	LIPUS (n = 14)	Historical control (n = 14)	P-value
Age, yr	67.3 ± 7.3	66.0 ± 9.6	0.678
Gender, men/women	11/3	11/3	>0.99
Body mass index, kg/m^2^	21.2 ± 3.6	23.9 ± 5.1	0.116
Rutherford category, n (%)			
4	3	1	
5	9	10	
6	2	5	
Medical history, n (%)			
Hypertension	9 (64)	14 (100)	0.041
Dyslipidemia	5 (36)	9 (64)	0.257
Diabetes mellitus	9 (64)	13 (93)	0.165
Previous myocardial infarction	9 (64)	8 (57)	>0.99
Previous stroke	4 (28)	3 (21)	>0.99
Chronic kidney disease	9 (64)	11 (78)	0.678
Hemodialysis	7 (50)	7 (50)	>0.99
Smoker (pre)	9 (64)	10 (71)	>0.99
Medications, n(%)			
Anti-coagulant	5 (36)	3 (21)	0.678
Anti-platelets	12 (86)	12 (86)	>0.99
RAS inhibitors	6 (43)	9 (64)	0.449
Calcium-channel blockers	1 (7)	3 (21)	0.596
Statins	3 (21)	6 (43)	0.420
Sulfonylurea/metformin/other	3 (21)	4 (28)	>0.99
Insulin	6 (43)	8 (57)	0.706

CLI indicates critical limb ischemia; LIPUS, low-intensity pulsed ultrasound; RAS, renin-angiotensin system.

### Overall major amputation-free survival rate after LIPUS

**Fig 2:**
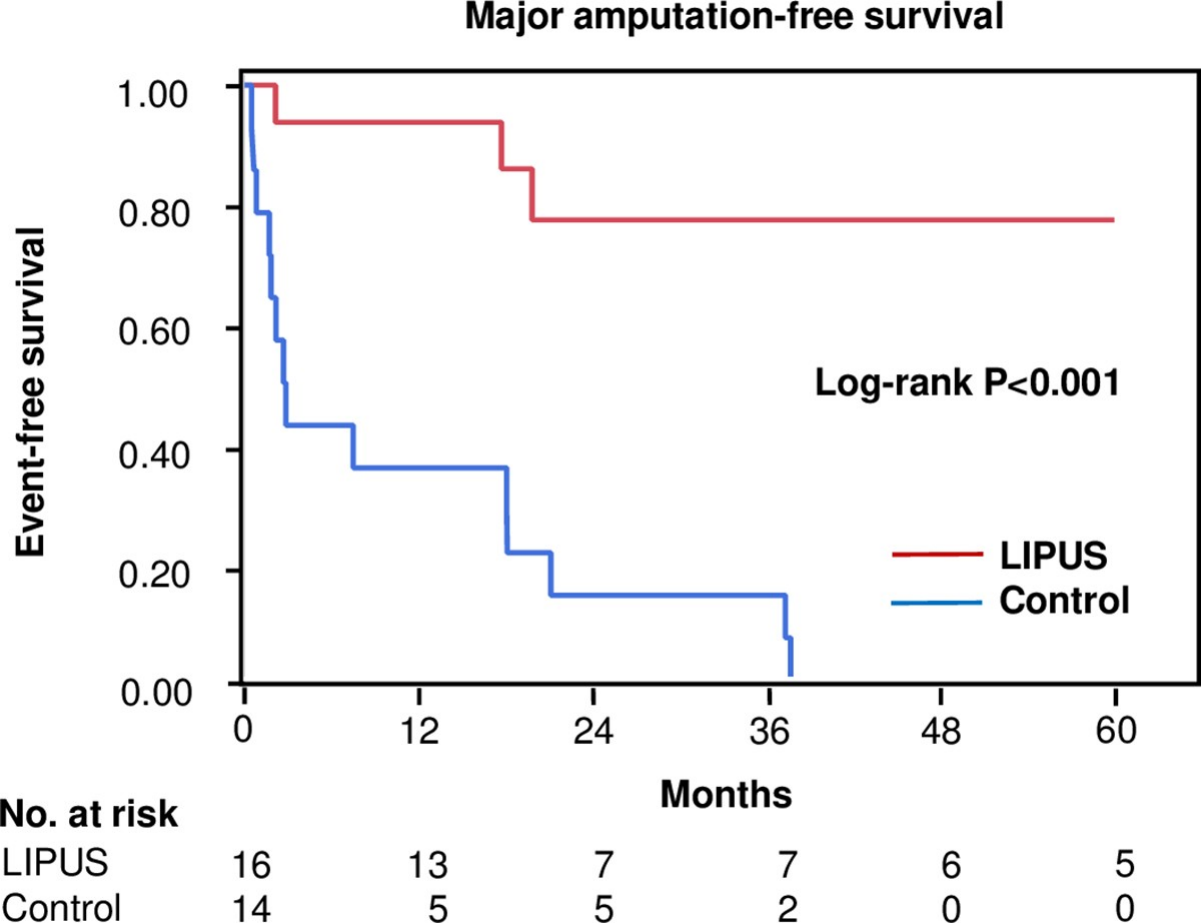
Kaplan-Meier analysis of major amputation-free survival rates in patients with CLI who were treated with LIPUS and in historical controls.

The mean duration of LIPUS exposure was 381± 283 days and the follow-up period was for 5 years after LIPUS exposure. After 5 years of follow-up, there were 3 major amputations in the LIPUS treatment group and 14 major amputations in the historical group. Kaplan-Meier analysis revealed the overall amputation-free survival rate was significantly higher in patients who were treated with LIPUS than in historical controls (Fig 2).

### Overall survival rate after LIPUS

**Fig 3:**
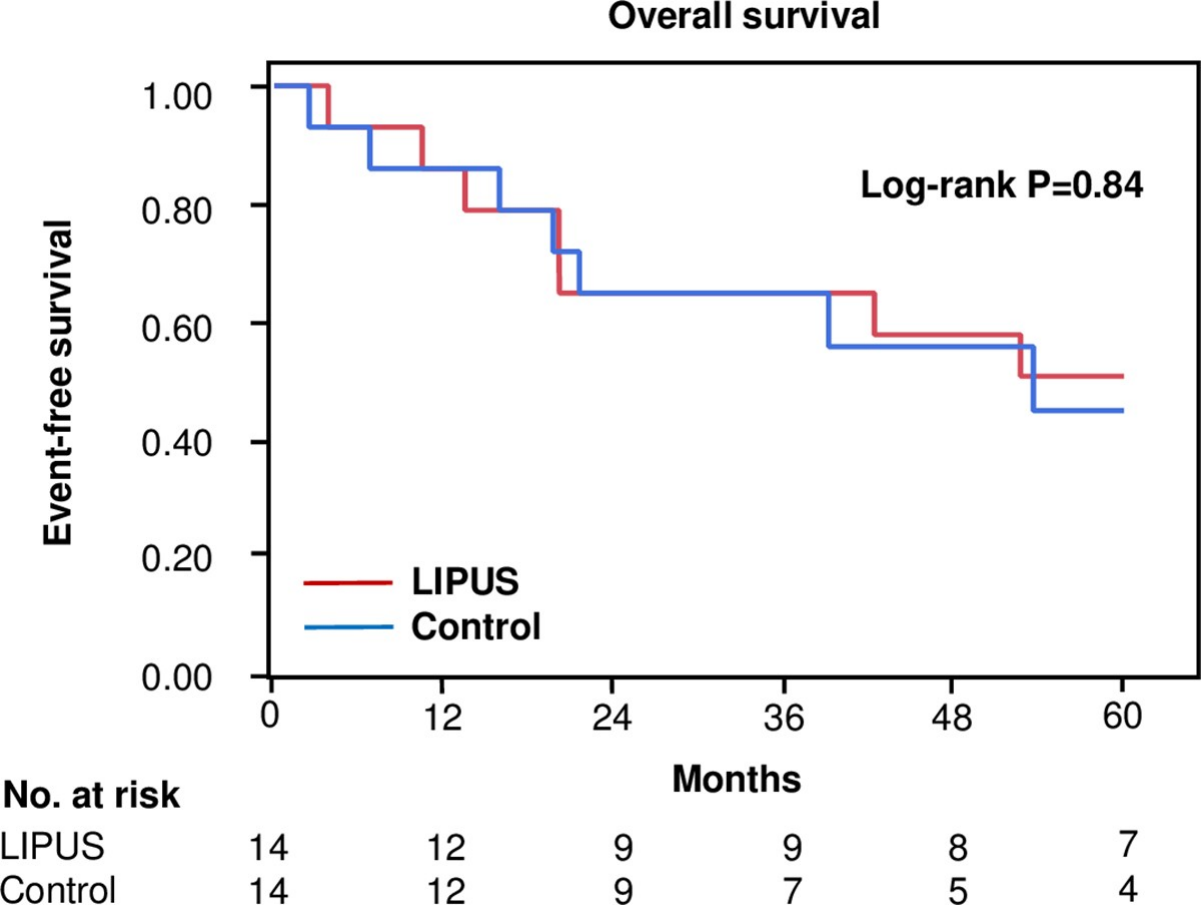
Kaplan-Meier analysis of overall survival rates in patients with CLI who were treated with LIPUS and in historical controls.

After 5 years of follow-up, there were 7 deaths in the LIPUS treatment group and 7 deaths in the historical group. There was no significant difference between the overall survival rates in the two groups (Fig 3). There was no report of malignancy during the study period. The causes of deaths in patients with CLI who underwent LIPUS exposure and historical controls up to 5 years after LIPUS exposure are shown in Table 2.

**Table 2:** Causes of death in patients with CLI who were treated with LIPUS and historical controls up to 5 years of follow-up.

All subjects	LIPUS (n = 14)	Historical control (n = 14)	P-value
Deaths, n	7	7	>0.99
Myocardial infarction, n	0	2	0.481
Heart failure, n	1	1	>0.99
Stroke, n	1	0	>0.99
Sepsis, n	1	2	>0.99
Malignancy, n	0	0	N. A.
Death from any cause, n	4	2	0.648

CLI indicates critical limb ischemia; LIPUS, low-intensity pulsed ultrasound.

### MACE after LIPUS

During the 5-year of follow-up period, 2 subjects died from cardiovascular causes, 1 had stroke, and 1 had hospitalization for heart failure in the LIPUS group and 3 subjects died from cardiovascular causes, 2 had myocardial infarction and 1 had stroke in the historical control group. There was no significant difference in 3-point MACE between patients with CLI who were treated with LIPUS and the historical group up to 5 years after LIPUS exposure.

### Acute adverse effects after LIPUS

There were no adverse effects and no severe acute adverse effects in patients with CLI who were treated with LIPUS.

## Discussion

In the present study, we demonstrated that patients who were treated with LIPUS had significantly better limb survival than that of historical controls for limbs that were deemed unsalvageable and/or deemed for amputation during a 5-year follow-up period. LIPUS did not alter the survival rate of those patients compared to that of historical controls in a similar period of time. There was no report of serious acute adverse effects within the study period.

It is clinically important to focus on the microvasculature and nonvascular tissue such as skeletal muscle and skin as well as re-establishing macrovascular blood flow in the management of CLI [18–20]. Degradation of skeletal muscle, skin and other tissue is associated with degradation of the vascular network. The use of microvascular regeneration strategies through direct tissue stimulation during the critical phase of the threatened limb together with optimized medical care would enable avoidance of major amputation in the long term. Therefore, the focus of therapeutic angiogenesis strategies has been not only on the macrovasculature but also on the need for concomitant microvascular regeneration along with skeletal muscle and related local tissue recovery from chronic damage.

In general practice, about one third of patients with CLI were not suitable for conventional intervention at the time of presentation [5]. The effectiveness of therapies needs to be weighed against the severity of the disease burdened by high morbidity and mortality rates. Research on treatment options and optimization of therapies are in progress to provide alternatives other than amputation for patients with severe CLI. There are various challenges in translating the established pre-clinical evidence into clinical practice [21,22]. To examine the long-term outcomes of the study, historical control data analysis along with updated studies will enable us to clarify the actual benefits of the studied treatment option. Therapeutic angiogenesis approaches by means of microvascular regeneration through cell therapy, gene therapy and LIPUS, the outcomes of which were compared with those of historical control subjects in our pilot trial, are being investigated to improve outcomes in patients with CLI.

It has been shown that LIPUS induces endothelial cell proliferation, migration and sprouting with increased capillary density and increased blood flow in LIPUS-treated ischemic hind-limb models [13–16]. The predetermined therapeutic range stimulates nitric oxide and prostaglandin E_2_ synthesis along with Ca^2+^ release to induce angiogenesis. Expression levels of VEGF, FGF2, angiopoietin 1 and transcriptional factor of PPAR gamma coactivator-1α (PGC-1α) were significantly increased in mesenchymal stroma/stem cells after LIPUS exposure. PGC-1α is known to play a role in the regulation of cellular energy metabolism. PGC-1α induced VEGF independently of the transcription factor hypoxia-induced factor pathway. LIPUS-induced angiogenesis may be due to mechanisms similar to those of exercise-induced angiogenesis through paracrine and metabolic effects [14]. LIPUS also induces phosphorylation of ERK1/2 and p38, members of the mitogen-activated protein kinase family, through activation of integrin, integrin receptors and Src and the activation of Rho/Rho-associated kinase/Src/ERK signaling pathway, leading to the promotion of cell proliferation [11–14]. LIPUS exposure should be one strategy for an increase in microvasculature in critical and chronic limb ischemia.

### Study limitations

This study has a number of limitations. It was performed in a single center with a small number of patients and is not a prospectively randomized trial. However, in the present study, we found a significant difference between major amputation rates in the LIPUS group and historical control group in a 5-year follow-up period. LIPUS exposure in patients with CLI was performed according to inclusion and exclusion criteria based on the registered clinical trial as mentioned in the method section, whereas data for historical controls were collected with no inclusion or exclusion criteria. The patients enrolled in this study and those in the historical control group were referred to the University Hospital Vascular Unit with severe disease at different times and were deemed unsuitable for conventional limb salvage treatments with multiple comorbidities. Future studies are needed to include more patients and to analyze the outcomes in different clinical settings and approaches.

## Conclusions

LIPUS is a noninvasive option for therapeutic angiogenesis with the potential to improve ischemic limb conditions in patients with CLI. Modulating the treatment approach to include several therapies for PAD including exercise, pharmacologic medication, inflow and outflow macrovascular interventions, and microvascular regeneration through cell and gene therapies along with LIPUS may improve overall outcomes in patients with PAD including CLI.

## References

[1] Farber A. Chronic limb-threatening ischemia. N Engl J Med. 2018; 379: 171–80. doi: 10.1056/NEJMcp1709326 29996085

[2] Norgren L, Hiatt WR, Dormandy JA, Nehler MR, Harris KA, Fowkes FGR, et al. Inter-society consensus for the management of peripheral arterial disease (TASC II). J Vasc Surg 2007; 45: 5–67. doi: 10.1016/j.jvs.2006.12.037 17223489

[3] Abola MTB, Golledge J, Miyata T, Rha SW, Yan BP, Dy TC, et. al. Asia-pacific consensus statement on the management of peripheral artery disease: a report from the asian pacific society of atherosclerosis and vascular disease asia-pacific peripheral artery disease consensus statement project committee. J Atheroscler Thromb 2020; 27: 809–907. doi: 10.5551/jat.53660 32624554 PMC7458790

[4] Hiatt WR. From the Masters: A sea-change for TransAtlantic Inter-Society Consensus (TASC). Vasc Med 2020; 5: 103–105. doi: 10.1177/1358863X20905651 32202476

[5] Elbadawi A, Elgendy IY, Saad M, Elzeneini M, Megaly M, Omer M, et. al. Contemporary revascularization strategies and outcomes among patients with diabetes with critical limb ischemia: insights from the national inpatient sample. JACC Cardiovasc Interv 2021; 14: 664–674. doi: 10.1016/j.jcin.2020.11.032 33640391

[6] Duff S, Mafilios MS, Bhounsule P, Hasegawa JT. The burden of critical limb ischemia: a review of recent literature. Vasc Health Risk Manag 2019; 15: 187–208. doi: 10.2147/VHRM.S209241 31308682 PMC6617560

[7] Masi S, Rizzoni D, Taddei S, Widmer RJ, Montezano AC, Lucher TF, et. al. Assessment and pathophysiology of microvascular disease: recent progress and clinical implications. Eur Heart J 2020; ehaa857. doi: 10.1093/eurheartj/ehaa857 33257973 PMC8266605

[8] Chevalier J, Yin H, Arpino JM, O’Neil C, Nong Z, Gilmore KJ, et. al. Obstruction of small arterioles in patients with critical limb ischemia due to partial endothelial- to-mesenchymal transition. iScience 2020; 23: 101251. doi: 10.1016/j.isci.2020.101251 32629616 PMC7322363

[9] Beckman JA, Duncan MS, Damrauer SM, Wells QS, Barnett JV, Wasserman DH, et. al. Microvascular disease, peripheral artery disease, and amputation. Circ 2019; 140: 449–458. doi: 10.1161/CIRCULATIONAHA.119.040672 31280589 PMC6682431

[10] Behroozian A, Beckman JA. Microvascular disease increases amputation in patients with peripheral artery disease. Arterioscler Thromb Vasc Biol 2020; 40:534–540. doi: 10.1161/ATVBAHA.119.312859 32075418

[11] Young SR, Dyson M. The effect of therapeutic ultrasound on angiogenesis. Ultrasound Med Biol 1990; 16: 261–269. doi: 10.1016/0301-5629(90)90005-w 1694604

[12] Suchkova VN, Baggs RB, Sahni SK, Francis CW. Ultrasound improves tissue perfusion in ischemic tissue through a nitric oxide dependent mechanism. Thromb Haemost 2002; 88: 865–870. 12428107

[13] Barzelai S, Sharabani-Yosef O, Holbova R, Castel D, Walden R, Engelberg S, et al. Low-intensity ultrasound induces angiogenesis in rat hind-limb ischemia. Ultrasound Med Biol 2006; 32: 139–45. doi: 10.1016/j.ultrasmedbio.2005.08.010 16364805

[14] Iwamoto A, Hidaka T, Kihara Y, Kubo H, Higashi Y. Low-Intensity Pulsed Ultrasound. In: Higashi Y., Murohara T. (eds) Therapeutic Angiogenesis. Springer, Singapore; 2017. pp. 161–175.

[15] Xavier CAM, Duarte LR. Estimulacao ultra-sonic de calo osseo: appicacao clinia. Rev Bras Ortop 1983; 18: 73–80.

[16] Heckman JD, Ryaby JP, McCabe J, Frey JJ, Kilcoyne RF. Acceleration of tibial fracture-healing by non-invasive, low-intensity pulsed ultrasound. J Bone Joint Surg Am 1994; 76: 26–34. doi: 10.2106/00004623-199401000-00004 8288661

[17] Kristiansen TK, Ryaby JP, McCabe J, Frey JJ, Roe LR. Accelerated healing of distal radial fractures with the use of specific, low-intensity ultrasound. A multicenter, prospective, randomized, double-blind, placebo-controlled study. J Bone Joint Surg Am 1997; 79: 961–973. doi: 10.2106/00004623-199707000-00002 9234872

[18] Mustapha JA, Anose BM, Martinsen BJ, Pliagas G, Ricotta J, Boyes CW et. al. Lower extremity revascularization via endovascular and surgical approaches: A systematic review with emphasis on combined inflow and outflow revascularization. SAGE Open Med 2020; 8: 2050312120929239. doi: 10.1177/2050312120929239 32551113 PMC7278295

[19] Kim K, Anderson EM, Scali ST, Ryan TE. Skeletal muscle mitochondrial dysfunction and oxidative stress in peripheral arterial disease: a unifying mechanism and therapeutic target. Antioxidants (Basel) 2020; 9: 1304. doi: 10.3390/antiox9121304 33353218 PMC7766400

[20] Latroche C, Gitiaux C, Chrétien F, Desguerre I, Mounier R, Chazaud B. Skeletal muscle microvasculature: a highly dynamic lifeline. Physiology (Bethesda) 2015; 30: 417–27. doi: 10.1152/physiol.00026.2015 26525341

[21] Yusoff FM, Kajikawa M, Matsui S, Hashimoto H, Kishimoto S, Maruhashi T, et al. Review of the long-term effects of autologous bone-marrow mononuclear cell implantation on clinical outcomes in patients with critical limb ischemia. Sci Rep 2019; 9: 7711. doi: 10.1038/s41598-019-44176-5 31118440 PMC6531470

[22] Fadini GP, Agostini C, Avogaro A. Autologous stem cell therapy for peripheral arterial disease meta-analysis and systematic review of the literature. Atherosclerosis 2010; 209: 10–17. doi: 10.1016/j.atherosclerosis.2009.08.033 19740466

